# Health-related quality of life of children born very preterm: a multinational European cohort study

**DOI:** 10.1007/s11136-022-03217-9

**Published:** 2022-08-17

**Authors:** Sung Wook Kim, Lazaros Andronis, Anna-Veera Seppänen, Adrien M. Aubert, Henrique Barros, Elizabeth S. Draper, Mariane Sentenac, Jennifer Zeitlin, Stavros Petrou, On Behalf of the SHIPS Research Group, J. Lebeer, P. Van Reempts, E. Bruneel, E. Cloet, A. Oostra, E. Ortibus, I. Sarrechia, K. Boerch, P. Pedersen, L. Toome, H. Varendi, M. Männamaa, P. Y. Ancel, A. Burguet, P. H. Jarreau, V. Pierrat, P. Truffert, R. F. Maier, M. Zemlin, B. Misselwitz, L. Wohlers, M. Cuttini, I. Croci, V. Carnielli, G. Ancora, G. Faldella, F. Ferrari, A. van Heijst, C. Koopman-Esseboom, J. Gadzinowski, J. Mazela, A. Montgomery, T. Pikuła, H. Barros, R. Costa, C. Rodrigues, U. Aden, E. S. Draper, A. Fenton, S. J. Johnson, S. Mader, N. Thiele, J. M. Pfeil, S. Petrou, S. W. Kim, L. Andronis, J. Zeitlin, A. M. Aubert, C. Bonnet, R. El Rafei, A. V. Seppänen

**Affiliations:** 1 Nuffield Department of Primary Care Health Sciences, University of Oxford, Radcliffe Observatory Quarter, Woodstock Road, Oxford, OX2 6GG UK; 2 Warwick Clinical Trials Unit, Warwick Medical School, University of Warwick, Coventry, UK; 3 Université Paris Cité, Inserm, INRAE, Centre for Research in Epidemiology and StatisticS (CRESS), Obstetrical Perinatal and Pediatric Epidemiology Research Team, EPOPé, 75004 Paris, France; 4 EPIUnit-Instituto de Saúde Pública da Universidade do Porto, Porto, Portugal; 5 Department of Health Sciences, University of Leicester, Leicester, UK

**Keywords:** Preterm birth; health-related quality of life, Mediation analysis, Children, PedsQL

## Abstract

**Purpose:**

This study aims to (1) describe the health-related quality of life (HRQoL) outcomes experienced by children born very preterm (28–31 weeks’ gestation) and extremely preterm (< 28 weeks’ gestation) at five years of age and (2) explore the mediation effects of bronchopulmonary dysplasia (BPD) and severe non-respiratory neonatal morbidity on those outcomes.

**Methods:**

This investigation was based on data for 3687 children born at < 32 weeks’ gestation that contributed to the EPICE and SHIPS studies conducted in 19 regions across 11 European countries. Descriptive statistics and multi-level ordinary linear squares (OLS) regression were used to explore the association between perinatal and sociodemographic characteristics and PedsQL^™^ GCS scores. A mediation analysis that applied generalised structural equation modelling explored the association between potential mediators and PedsQL^™^ GCS scores.

**Results:**

The multi-level OLS regression (fully adjusted model) revealed that birth at < 26 weeks’ gestation, BPD status and experience of severe non-respiratory morbidity were associated with mean decrements in the total PedsQL^™^ GCS score of 0.35, 3.71 and 5.87, respectively. The mediation analysis revealed that the indirect effects of BPD and severe non-respiratory morbidity on the total PedsQL^™^ GCS score translated into decrements of 1.73 and 17.56, respectively, at < 26 weeks’ gestation; 0.99 and 10.95, respectively, at 26–27 weeks’ gestation; and 0.34 and 4.80, respectively, at 28–29 weeks’ gestation (referent: birth at 30–31 weeks’ gestation).

**Conclusion:**

The findings suggest that HRQoL is particularly impaired by extremely preterm birth and the concomitant complications of preterm birth such as BPD and severe non-respiratory morbidity.

**Supplementary Information:**

The online version contains supplementary material available at 10.1007/s11136-022-03217-9.

## Plain English summary

Preterm birth is associated with an increased risk of mortality in comparison to birth at term. Compared to children born at term, preterm born children are more likely to develop serious complications such as bronchopulmonary dysplasia (BPD) and severe non-respiratory morbidity, including intraventricular haemorrhage, periventricular leukomalacia, necrotising enterocolitis and retinopathy of prematurity. Nevertheless, evidence on the mechanism through which effects of neonatal morbidities are transmitted to the health-related quality of life (HRQoL) of those born preterm is scarce. Hence, this study aims to investigate the mechanism. Furthermore, this study aims to describe the HRQoL outcomes experienced by children born preterm at five years of age. The study used data for 3687 children obtained from the EPICE (Effective Perinatal Intensive care in Europe) and SHIPS (Screening to improve Health In very Preterm infantS) studies. The data were collected from 11 European countries. Data were analysed using a multi-level ordinary least squares and a separate mediation analysis. The new evidence provided by this study shows that neonatal morbidities and, in particular severe non-respiratory morbidity, are the main drivers of HRQoL outcomes in children born very preterm and extremely preterm. These results can be used to inform service targeting towards these higher risk groups.

## Introduction

Very preterm birth (VPT, birth at 28 to 31 weeks’ gestation) and extremely preterm birth (EPT, birth at < 28 weeks’ gestation) are associated with an increased risk of mortality in comparison to birth at term [1]. Moreover, although survival rates following VPT and EPT have increased in recent years [2], surviving children are at increased risk of cerebral palsy, visual and auditory deficits, poor respiratory outcomes, impaired motor and cognitive ability and psychiatric disorders compared to children born at term [3–6]. Furthermore, up to one third of children born VPT or EPT and their parents face a life course with significant morbidity, dependency, low educational achievement and socioeconomic challenges [7–9].

There is increasing recognition of the need to measure the impact of preterm birth across multiple domains [10]. Assessments of health-related quality of life (HRQoL) outcomes provide valuable complementary data to biomedical assessments that have traditionally been reported [10]. Importantly, they also provide a framework for reflecting the multi-dimensional aspects of physical, mental and social well-being of those born preterm and their families. Several assessments of HRQoL following preterm birth have applied multi-dimensional health profile measures that disaggregate outcomes across dimensions or subscales [11–13]. A small number of recent assessments of the HRQoL of preterm-born children [14–16] have applied the Pediatric Quality of life Inventory^™^ (PedsQL^™^) [17], which was designed to provide an age-specific modular approach to measuring HRQoL in healthy children and adolescents, as well as those with acute and chronic health conditions, across the broadest empirically feasible age groups. However, these studies are based on case series or cohorts with relatively small samples, with each limited to application in one country.

It is well known that, compared to children born at term, preterm born children are more likely to develop serious complications such as bronchopulmonary dysplasia (BPD) [18, 19] and severe non-respiratory morbidity [20–22]. Those who develop BPD can suffer from long-term respiratory complications, such as impaired lung function, which can have life-long consequences for those born preterm [18]. Likewise, the severe non-respiratory morbidity associated with preterm birth can also result in decrements in the HRQoL of those born preterm. For example, one study found that pre-school children born preterm who had been admitted to neonatal intensive care due to morbidity associated with preterm birth had significantly poorer HRQoL than children born at term across the measurement scales for lungs, stomach, eating disorders, motor functioning and communication of the TNO-AZL Preschool Quality Of Life questionnaire [23]. However, evidence on the mediation effects of BPD and severe non-respiratory morbidity on the HRQoL of those born preterm is scarce. More generally, studies are required to understand the mechanisms through which preterm birth affects the HRQoL of those born very or extremely preterm. The aims of this study are therefore two-fold: firstly, to describe the HRQoL experienced by children born VPT or EPT across multi-dimensional outcomes and, secondly, to explore the mediation effects of BPD and severe non-respiratory morbidity on those outcomes.

## Methods

### Study population

Data for this study were obtained from the EPICE (Effective Perinatal Intensive care in Europe) cohort study and SHIPS (Screening to improve Health In very Preterm infantS), a follow-up study for EPICE. A detailed description of the design, outcome measures and data collection processes in the EPICE and SHIPS studies can be found elsewhere [24]. In brief, the EPICE and SHIPS studies constituted and followed up a population-based prospective cohort of all children born between 22^+0^ weeks and 31^+6^ weeks of gestation over a 12-month period (except in France where it was conducted over a 6-month period) in 2011–2012 in 19 regions across 11 European countries: Belgium (Flanders); Denmark (Eastern Region); Estonia (entire country); France (Burgundy, Ile-de-France and the Northern region); Germany (Hesse and Saarland); Italy (Emilia-Romagna, Lazio and Marche); the Netherlands (Central and Eastern region), Poland (Wielkopolska); Portugal (Lisbon and Northern region); Sweden (Greater Stockholm) and the United Kingdom (UK) (East Midlands, Northern and Yorkshire & the Humber regions). Perinatal data were extracted from medical records whilst parental questionnaires at two and five years of age were used as the primary vehicle for assessments of longer term outcomes.

### Health-related quality of life

To measure HRQoL outcomes, the PedsQL^™^ 4.0 Generic Core Scales (hereafter PedsQL GCS for brevity) were completed via postal questionnaires by parents when their children were five years of age. The parent proxy-report [ages 5–7 (young child)] format was used. The PedsQL GCS have been developed to measure the core dimensions of health as well as school functioning [25]. The PedsQL GCS contain 23 items covering physical functioning (8 items), emotional functioning (5 items), social functioning (5 items) and school/day care functioning (5 items). A 5-point response scale is utilised across each item (0 = never a problem; 1 = almost never a problem; 2 = sometimes a problem; 3 = often a problem; 4 = almost always a problem). Raw responses were scored (0 to 4) and reversely transformed to a 0–100 scale. The mean of scale scores was computed as the sum across the relevant items over the number of items answered, thereby accounting for missing data [25]. The mean psychosocial health summary score was calculated as the sum of the item scores over the number of the items completed for the emotional, social and school functioning scales, whilst the physical health summary score was identical to the physical functioning scale score. The total score represents the sum of scores across all 23 items divided by the number of items answered. Higher scores indicate better HRQoL.

### Predictive factors

The key predictive factors incorporated into the analysis that explored mediation effects were gestational age at birth, BPD status and severe non-respiratory morbidity status, all of which were measured using data extracted from medical records. Gestational age at birth was classified into four categories of < 26 weeks, 26 to 27 weeks, 28 to 29 weeks and 30 to 31 weeks. BPD was defined as receipt of supplemental oxygen and/or ventilatory support (continuous positive airway pressure (CPAP) or mechanical ventilation) at 36 weeks of postmenstrual age and dichotomised by its presence or absence. Severe non-respiratory morbidity consisted of intraventricular haemorrhage (IVH) grade III/IV, cystic periventricular leukomalacia (cPVL), retinopathy of prematurity (ROP) stage III or more or necrotizing enterocolitis (NEC) requiring surgery or peritoneal drainage and dichotomised according to the presence or absence of at least one of these morbidities.

### Statistical analysis

Differences in the total PedsQL GCS score in relation to preterm status (VPT vs EPT), and presence of BPD (no, yes) or severe non-respiratory morbidity (no, yes), were tested using Student’s *t* test. These differences were estimated for each of the scales of the PedsQL GCS, namely physical functioning, emotional functioning, social functioning, school functioning, psychosocial functioning, as well as for the total score.

Multi-level regression analysis was initially carried out to explore the association between the total PedsQL GCS score and perinatal, neonatal and sociodemographic variables. As the overall PedsQL GCS score is a continuous variable, ordinary least square (OLS) regression was employed in the analysis. The following levels of the multi-level analysis were specified: 1) individual child, 2) parent/caregiver and 3) country. Independent variables in the model were chosen based on published evidence [26–28], including published empirical models using data from the EPICE and SHIPS studies [29]. These included the following: gestational age at birth (weeks) (< 26, 26–27, 28–29, 30–31) [30], small for gestational age (SGA) status (birth weight < 10th, ≥ 10th centile for gestational age) [31], congenital anomaly status (no or yes), sex (male or female), multiplicity (singletons, twins or more, based on known associations between multiplicity and increased risk of preterm birth) [30], BPD status (no or yes), severe non-respiratory morbidity status (no or yes), highest maternal education level (high school or less, more than high school) [27], maternal country of birth (native-born, non-native from other European country, non-native from non-European country), maternal age at childbirth (years) (< 25, 25 to 34, > 34) and parity (primiparous, multiparous).

Two regression models were constructed for the multi-level OLS regression: (1) a partially adjusted model that excluded BPD status and severe non-respiratory morbidity status and (2) a fully adjusted model that included all independent variables. Due to different education systems across the participating countries with children starting compulsory schooling at different ages, regions in Denmark and Germany provided a low number of responses to questions about school functioning within the PedsQL GCS. To address this issue, a sensitivity analysis excluding Denmark and Germany was performed.

**Fig. 1:**
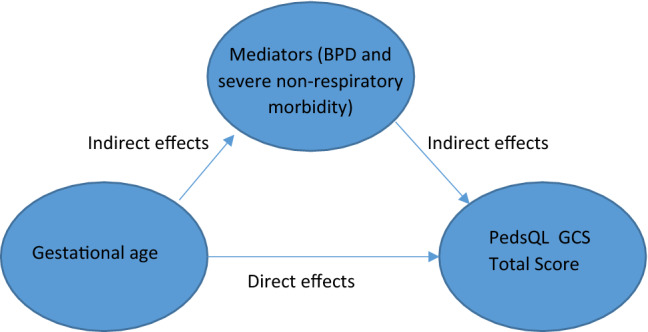
Overview of mediation analysis

BPD and severe non-respiratory morbidity can act as mediators that negatively affect health for preterm born children [28, 32]. Mediation analysis is a widely used tool for exploring whether the effect of an independent variable on a dependent variable is entirely or partially due to one or more intermediary (mediator) variables [33, 34]. In mediation analysis, the overall effect can be decomposed into direct and indirect effects. Total effects are calculated as the sum of the direct effects and indirect effects that are the product of path coefficients (e.g. preterm birth to BPD * BPD to PedsQL GCS score). This can be used to understand a relationship between an independent variable (*X*) and a dependent variable (*Y*) with the underlying mechanism that affects the dependent variable through a mediator variable (*M*). Simply adjusting for the intermediate variable can cause bias [35]. As a result, in a regression analysis, the coefficient of *X* on *Y* can be considered a direct effect, whereas the coefficient of *M* on *Y* can be considered an indirect effect. In cases where there are multiple mediators in the model, these can be estimated using either Structural Equation Modelling (SEM) or Generalised Structural Equation Modelling (GSEM) [36]. Direct effects indicate the effects of independent variables on a dependent variable whilst indirect effects indicate the effects that are mediated through the mediators on the dependent variable (Fig. 1). In general, if the indirect effects are statistically significant, it is believed that mediation effects exist [37]. In other words, the significance of the total effect is not necessary for the existence of mediation effects. In this study, the mediating variables were BPD and severe non-respiratory morbidity. Direct effects were estimated as the effects of gestational age per se on the total PedsQL GCS score whilst indirect effects were estimated as the effects of the mediating variables of BPD and severe non-respiratory morbidity on the total PedsQL GCS score. This is illustrated in Fig. 1. The GSEM framework was applied and standard errors were estimated using a bootstrap procedure run over 1000 iterations [38]. A sensitivity analysis that excluded data from Denmark and Germany was also conducted. Statistical analyses were carried out with using Stata 16.0 (StataCorp, College Station, TX).

## Results

Table 1 summarises the characteristics of the study children at birth, split by country. Estonia and the UK had the lowest (*N* = 153) and highest numbers (*N* = 1745) of preterm born study children, respectively. The number of children followed up at five years, using the denominator of all live and eligible children (*N* = 6759), was 3687 (54.5%). Of the eligible children, singletons represented 5437 (80.4%) of the preterm born children, whereas twins and higher order births represented 1322 (19.6%) of the preterm born children. Estonia had the highest follow-up rate (88%) whilst the UK had the lowest follow-up rate (26%).

**Table 1:** Descriptive statistics of participant characteristics (entire cohort of live births), by country

	Belgium	Denmark	Estonia	France	Germany	Italy	Netherlands	Poland	Portugal	UK	Sweden
Infants (*N*)	752	351	153	1307	758	1134	393	316	724	1745	267
Gestational age, completed weeks, Mean (SD)	28.6 (2.3)	28.0 (2.4)	28.7 (2.2)	28.4 (2.3)	28.2 (2.6)	28.5 (2.4)	28.4 (2.5)	28.1 (2.8)	28.5 (2.2)	28.5 (2.4)	28.2 (2.5)
Male, *N* (%)	426 (56.6)	185 (52.7)	80 (52.3)	701 (53.6)	407 (53.7)	563 (49.6)	195 (49.6)	176 (55.7)	423 (58.4)	965 (55.3)	145 (54.3)
Singleton, *N* (%)	464 (61.7)	208 (59.3)	119 (77.8)	881 (67.4)	487 (64.2)	768 (67.7)	274 (69.7)	251 (79.4)	500 (69.1)	1300 (74.6)	185 (69.3)
Twins or more, *N* (%)	288 (38.3)	143 (40.7)	34 (22.2)	426 (32.6)	271 (35.8)	366 (32.3)	119 (30.3)	65 (20.6)	224 (30.9)	443 (25.4)	82 (30.7)
Birth weight (grams), Mean (SD)	1260.0 (420.8)	1141.6 (415.4)	1287.0 (390.1)	1162.3 (372.1)	1165.1 (418.0)	1195.4 (420.3)	1198.2 (412.1)	1224.7 (474.2)	1149.4 (363.5)	1210.4 (383.9)	1216.7 (436.4)
Follow up at five years of age, *N* (%)	280 (37.2)	152 (43.3)	134 (87.6)	779 (59.6)	280 (36.9)	693 (61.1)	155 (39.4)	189 (59.8)	433 (59.8)	448 (25.7)	144 (53.9)

*UK* denotes United Kingdom; *SD* denotes standard deviation

Table 2 presents differences in mean PedsQL GCS scores by preterm status, BPD status and severe non-respiratory morbidity status. The results show that the total PedsQL GCS score differed significantly by preterm status (78.61 for VPT vs 75.70 for EPT; *p* < 0.001), BPD status (78.60 for no BPD vs 72.70 for BPD; *p* < 0.001) and severe non-respiratory morbidity status (78.59 for no morbidity vs 71.98 for morbidity; *p* < 0.001). Significant differences were also observed in PedsQL GCS physical, emotional, social, school and psychosocial functioning scores by preterm status, BPD status and severe non-respiratory morbidity status. A table that replicates these analyses for the four gestational age categories (< 26, 26–27, 28–29, 30–31 weeks) is presented in Online Appendix 1.

**Table 2:** Differences in PedsQL GCS scores by preterm, BPD and severe non-respiratory morbidity status

Preterm status	VPT, mean (SD)	EPT, mean (SD)	Mean difference	95% Confidence interval	*p* value
Physical functioning	81.34 (20.39)	77.82 (22.40)	3.41	(1.69, 5.13)	< 0.001
Emotional functioning	76.19 (16.70)	74.83 (17.77)	1.09	(− 0.30, 2.48)	0.037
Social functioning	82.74 (18.73)	79.03 (20.20)	3.26	(1.71, 4.81)	< 0.001
School functioning	76.51 (19.27)	72.91 (19.66)	3.64	(2.06, 5.21)	< 0.001
Psychosocial functioning	79.33 (14.90)	76.27 (16.19)	2.66	(1.60, 4.10)	< 0.001
Total score	78.61 (14.93)	75.70 (16.06)	2.85	(1.42, 3.90)	< 0.001

BPD	No, mean (SD)	Yes, mean (SD)	Mean difference	95% Confidence interval	*p* value
Physical functioning	81.27 (20.33)	74.41 (24.40)	6.86	(4.86, 8.86)	< 0.001
Emotional functioning	76.15 (16.65)	73.23 (18.91)	2.92	(1.29, 4.55)	0.001
Social functioning	82.76 (18.51)	75.14 (21.87)	7.62	(5.79, 9.45)	< 0.001
School functioning	76.61 (18.85)	69.10 (21.34)	7.51	(5.54, 9.48)	< 0.001
Psychosocial functioning	79.31 (14.78)	72.97 (17.99)	6.35	(4.89, 7.80)	< 0.001
Total score	78.60 (14.79)	72.70 (17.26)	5.90	(4.45, 7.35)	< 0.001

Severe non-respiratory morbidity	No, mean (SD)	Yes, mean (SD)	Mean difference	95% Confidence interval	*p* value
Physical functioning	81.52 (19.84)	71.43 (26.85)	10.1	(7.83, 12.37)	< 0.001
Emotional functioning	76.24 (16.85)	73.32 (17.59)	2.93	(1.06, 4.79)	0.002
Social functioning	82.70 (18.64)	74.20 (21.74)	8.51	(6.41, 10.60)	< 0.001
School functioning	76.44 (18.89)	68.04 (21.95)	8.40	(6.14, 10.67)	< 0.001
Psychosocial functioning	79.37 (14.70)	71.83 (18.00)	7.54	(5.88, 9.19)	< 0.001
Total score	78.59 (14.84)	71.98 (17.38)	6.62	(4.96, 8.28)	< 0.001

Differences in characteristics between preterm status, BPD and severe non-respiratory morbidity status were tested using the Student *t* test

*BPD* denotes bronchopulmonary dysplasia; *VPT* denotes very preterm; *EPT* denotes extremely preterm

Table 3 shows the results of the OLS regression based on multi-level modelling. Both partially and fully adjusted models present similar patterns in terms of the direction of signs, statistical significance and magnitude of coefficients. In the fully adjusted model, the total PedsQL GCS score of the children who were born at < 26 gestational weeks, with BPD or with severe non-respiratory morbidity were lower than the respective values for the reference group of children born at 30–31 gestational weeks, without BPD or without severe non-respiratory morbidity by 0.35 (*p* = 0.834), 3.71 (*p* < 0.001) and 5.87 (*p* < 0.001), respectively. Birth earlier than 26 weeks’ gestation was associated with a significant reduction in the total PedsQL GCS score in the partially adjusted model (*p* < 0.05), but not following further adjustment for BPD status and severe non-respiratory morbidity status. In addition, congenital anomaly status, sex, maternal education and maternal country of birth all had statistically significant effects in both the partially and fully adjusted models. For instance, in the fully adjusted model, the coefficients for presence of a congenital anomaly, male sex, highest maternal education of high school or less and non-European maternal country of birth were − 3.34 (*p* < 0.001), − 2.86 (*p* < 0.001), − 1.56 (*p* < 0.05) and − 6.45 (*p* < 0.001), respectively. The results of the sensitivity analysis excluding Denmark and Germany showed a similar pattern and are presented in Online Appendix 2. The results of a multi-level OLS regression that treated gestational age as a continuous variable is presented in Online Appendix 3.

**Table 3:** Multi-level regression analysis of the association between the total PedsQL GCS score and clinical and sociodemographic factors

			Partially adjusted^+^	Fully adjusted
Gestational age (weeks) (ref: 30–31)	< 26		− 4.46 (2.22)*	− 0.35 (1.64)
	26–27		− 1.46 (0.83)	0.42 (0.71)
	28–29		− 0.68 (0.89)	− 0.23 (0.82)
SGA (ref: ≥ 10th centile)	< 10th centile		− 0.74 (0.60)	− 0.39 (0.57)
Congenital anomalies (ref: no)	Yes		− 3.95 (0.87)***	− 3.34 (0.90) ***
Sex (ref: female)	Male		− 2.93 (0.69)***	− 2.86 (0.69) ***
Multiplicity (ref: singleton)	Twins and higher		2.02 (0.83)*	1.83 (0.87) *
BPD (ref: no)	Yes			− 3.71 (0.92) ***
Severe non-respiratory morbidity (ref: no)	Yes			− 5.87 (1.13) ***
Mother’s education (ref: higher education)	High school or less		− 1.71 (0.62)**	− 1.56 (0.66)*
Country of birth for mothers (ref: native)	Non-native, European born		− 0.18 (1.24)	0.59 (1.29)
	Non-native, non-European born		− 5.81 (0.99)***	− 6.45 (1.02)***
Mother’s age at childbirth (years) (ref: 25–34)	< 25		− 1.07 (0.99)	− 0.78 (0.95)
	> 34		1.31 (1.03)	1.33 (1.09)
Parity (ref: multiparous)	Primiparous		− 0.26 (0.65)	− 0.20 (0.55)

^+^Partially adjusted model excludes BPD and severe non-respiratory morbidity

*SGA* small for gestational age, *BPD* bronchopulmonary dysplasia. This is defined as receipt of supplemental oxygen and/or ventilatory support (CPAP or mechanical ventilation) at 36 weeks of postmenstrual age

Severe non-respiratory morbidity: Intraventricular haemorrhage grades III–IV (IVH), periventricular leukomalacia (PVL), retinopathy of prematurity stages III-V (ROP) or necrotising enterocolitis needing surgery (NEC)

Likelihood Ratio (LR) test for linear regression = *p* < 0.001

*p < 0.05, **p < 0.01, ***p < 0.001

**Fig. 2:**
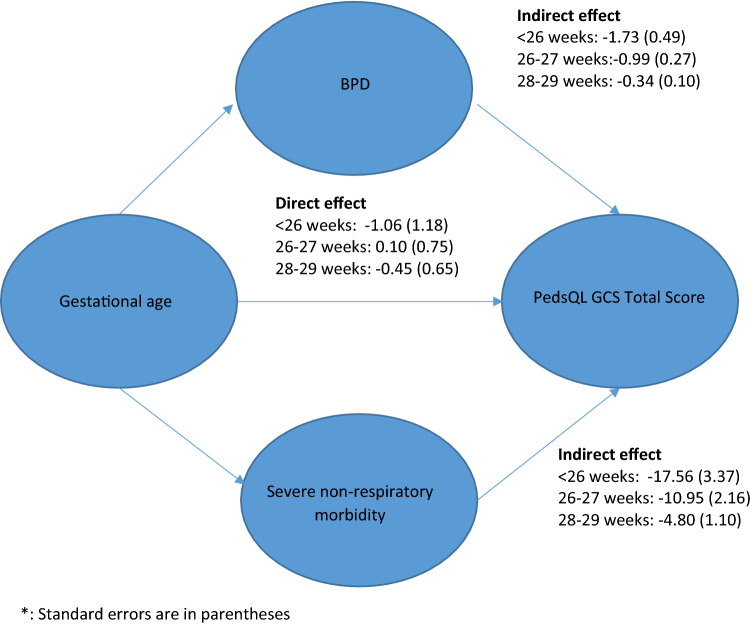
Mediation analysis of the indirect effects and total effects of preterm status on the total PedsQL GCS score^*^

Table 4 shows the results of the mediation analysis using BPD and severe non-respiratory morbidity as mediators based on the GSEM (Online Appendix 4). The indirect effects of BPD and severe non-respiratory morbidity on the total PedsQL GCS score were estimated at − 1.73 (bootstrap 95% confidence interval (CI) − 2.68 to − 0.77) and − 17.56 (bootstrap CI − 24.15 to − 10.96), respectively, at < 26 weeks’ gestation (referent: birth at 30–31 weeks’ gestation). The indirect effects of BPD and severe non-respiratory morbidity on the total PedsQL GCS score were estimated at − 0.99 (bootstrap 95% CI − 1.53 to − 0.46) and − 10.95 (bootstrap 95% CI − 15.19 to − 6.70), respectively, at 26–27 weeks’ gestation, and − 0.34 (bootstrap 95% CI − 0.54 to − 0.15) and − 4.80 (bootstrap 95% CI − 6.96 to − 2.64), respectively, at 28–29 weeks’ gestation (referent: birth at 30–31 weeks’ gestation). In contrast, the direct effects of birth at < 26 weeks, 26–27 weeks and 28–29 weeks were − 1.06 (bootstrap 95% CI − 3.38 to 1.26), 0.10 (bootstrap 95% CI − 1.37 to 1.57) and − 0.45 (bootstrap 95% CI − 1.73 to 0.82), respectively (referent: birth at 30–31 weeks’ gestation), which were not statistically significant. In short, Table 4 illustrates the importance of the indirect effects of BPD and severe non-respiratory morbidity on the total PedsQL GCS score, with diminishing indirect effects with increasing gestational age. This result is graphically presented in Fig. 2. Online Appendix 5 presents the sensitivity analysis that excluded Denmark and Germany from the mediation analysis and reveal similar magnitude of coefficients observed when the data for the entire study population were analysed.

**Table 4:** Mediation analysis of the indirect effects and total effects of preterm status on the total PedsQL GCS score (four categories of gestational age: < 26 weeks, 26–27 weeks; 28–29 weeks; 30–31 weeks (referent))

		Coef (SE)	Bootstrap 95% confidence interval
< 26	BPD: indirect effects^a^	− 1.73 (0.49)	(− 2.68, − 0.77)
	Direct effects^b^	− 1.06 (1.18)	(− 3.38, 1.26)
	BPD: total effects^c^	− 2.79 (1.16)	(− 5.06, − 0.51)
	Severe non-respiratory morbidity: indirect effects^a^	− 17.56 (3.37)	(− 24.15, − 10.96)
	Direct effects^b^	− 1.06 (1.18)	(− 3.38, 1.26)
	Severe non-respiratory morbidity: total effects^c^	− 18.62 (3.29)	(− 25.06, − 12.17)
26–27	BPD: indirect effects^a^	− 0.99 (0.27)	(− 1.53, − 0.46)
	Direct effects^b^	0.10 (0.75)	(− 1.37, 1.57)
	BPD: total effects^c^	− 0.90 (0.75)	(− 2.37, 0.58)
	Severe non-respiratory morbidity: indirect effects^a^	− 10.95 (2.16)	(− 15.19, − 6.70)
	Direct effects^b^	0.10 (0.75)	(− 1.37, 1.57)
	Severe non-respiratory morbidity: total effects^c^	− 10.85 (2.17)	(− 15.09, − 6.60)
28–29	BPD: indirect effects^a^	− 0.34 (0.10)	(− 0.54, − 0.15)
	Direct effects^b^	− 0.45 (0.65)	(− 1.73, 0.82)
	BPD: total effects^c^	− 0.80 (0.65)	(− 2.08, 0.49)
	Severe non-respiratory morbidity: indirect effects^a^	− 4.80 (1.10)	(− 6.96, − 2.64)
	Direct effects^b^	− 0.45 (0.65)	(− 1.73, 0.82)
	Severe non-respiratory morbidity: total effects^c^	− 5.25 (1.24)	(− 7.68, − 2.82)

This was performed based on 1000 bootstrap simulations

^a^This presents the indirect effect of each mediator (BPD and Severe non-respiratory morbidity) on the PedsQL GCS score. See Fig. 1

^b^This represents the direct effect of gestational age (GA) on the PedsQL GCS score. See Fig. 1

^c^Total effects are calculated as the sum of the direct effects and indirect effects that are the product of path coefficients (e.g. preterm birth to BPD * BPD to PedsQL GCS score)

## Discussion

This study provides new evidence on HRQoL outcomes in mid-childhood of children born very or extremely preterm in 11 European countries. To the best of our knowledge, this is the first study that provides information with respect to HRQoL outcomes, as measured by the PedsQL GCS, of children born preterm across several European countries. The estimated PedsQL GCS scores are based on 3687 VPT born children in Europe. In addition, this study explores the mediation effects of BPD and severe non-respiratory morbidity on PedsQL GCS scores.

The study confirms that the HRQoL of VPT born children is significantly better in those who did not experience BPD or severe non-respiratory morbidity. This result is consistent with evidence from existing studies [28, 39, 40]. A study conducted in the USA [41] reported that in children who survived to 18–36 months of corrected age with severe BPD, the mean physical health summary score of the PedsQL GCS was 78.0 and the mean psychosocial health summary score of the PedsQL GCS was 75.3. This compared with physical health and psychosocial health summary scores of 74.4 and 73.0, respectively, observed amongst children with BPD in our study (descriptive analyses). Although it is not straightforward to compare and contrast our PedsQL GCS score findings with those in other studies and with ‘population’ norms, a study conducted in Canada [42] for liver transplant children aged under 6 found that healthy children had a mean total PedsQL score of 82, compared to 73 and 75 for chronically ill children and liver transplant children, respectively.

It is unclear whether the approximate 3 point difference in mean total PedsQL GCS that we observed between children born VPT and EPT can be considered clinically meaningful to stakeholders as there is, to our knowledge, no generally accepted minimum clinically important difference in the total PedsQL GCS score. However, there have been a few studies that have generated estimates of minimally important clinical differences for the PedsQL GCS score [42–44]. Amongst these studies, a US study [43] that estimated PedsQL GCS scores of 20,031 families with children aged 2–16 years showed that healthy children had a mean total PedsQL GCS score of 84, compared to 74 for chronically ill children. Furthermore, as noted above, a Canadian study [42] of liver transplant children aged under 6 years found that healthy children had a mean total PedsQL GCS score of 82, compared to 73 and 75 for chronically ill children and liver transplant children, respectively.

In our partially adjusted multi-level OLS model, birth at < 26 weeks’ gestation was associated with a statistically significant decrement in HRQoL, consistent with evidence from other research studies [40, 45, 46], but this effect dissipated after adjustment for BPD status and severe non-respiratory morbidity status (fully adjusted model). Our multi-level OLS model also found that presence of a congenital anomaly, male sex, low maternal education status and non-European maternal country of birth are independent predictors of the HRQoL of VPT born children. Comparative evidence of the association between these factors and the HRQoL of VPT born children is sparse. One study conducted in Finland found that presence of a congenital anomaly was negatively associated with the HRQoL of preterm born children born between 2000 and 2003 [47]. A study of early-school age children born preterm, conducted in France, revealed that poorer HRQoL was reported for boys than for girls when the ‘Vécu et Santé Perçue de l’Adolescent et de l’Enfant’ (VSP-A) questionnaire (parent version) was used, but the difference was not statistically significant [48]. Low parental education status was also negatively associated with the HRQoL of preterm born children in a Taiwanese study, but the association was not statistically significant [49]. We are not, however, aware of any other studies that have explored the association between maternal country of birth and the HRQoL of preterm born children, although the association between migrant status and preterm birth rates is well established [50–52].

This study provides clear evidence around the mediation effects of BPD and severe non-respiratory morbidity on the HRQoL outcomes of VPT born children in European countries. Although there have been numerous studies describing the negative effects of perinatal complications on HRQoL outcomes, the mechanisms through which preterm birth translates into poorer HRQoL have not been elucidated. Existing studies [32, 41, 53] focus on either the association between complications and HRQoL outcomes or the association between preterm birth per se and HRQoL outcomes [40] rather than exploring or focussing on the mediating mechanisms through which those outcomes are experienced.

The mediation analysis performed in this study shows that an association exists between the complications associated with preterm birth including BPD and, in particular, severe non-respiratory morbidity which explains a high proportion of the total effect of gestational age and total PedsQL GCS scores. It was found that gestational age at birth no longer had a continued direct effect on outcomes after these indirect effects were taken into consideration as the direct effects of gestational age were not statistically significant. This implies that poorer HRQoL as measured by the total PedsQL GCS score is primarily driven by the complications of preterm birth rather than preterm status per se. The negative effects of BPD and severe non-respiratory morbidity for the health and development of preterm born children have been well reported, with severe non-respiratory morbidities, namely brain lesions, being strongly associated with some of the most severe impairments, such as cerebral palsy [54]. Further, one study reported that, within a preterm group, infants who had neonatal steroid treatment for BPD presented lower HRQoL scores as measured by the Child Health Questionnaire (CHQ-Pf 50) than infants who did not have this treatment [40].

Mediation analysis is, in principle, a causal inference model [55]. However, a mediation analysis proposes the causal relationship between the independent variable and the dependent variable by mediating variables rather than directly showing the causal relationship between the independent variable and the dependent variable. It has been argued that statistical significance only confirms the effect that the presumed model provides [56]. The strength of a mediation analysis comes from the appropriate study design based on the clinical and epidemiological context. Given this, the results of this study should be carefully interpreted because mediation is not defined statistically.

The main strength of this study comes from its large sample size of approximately 3600 born very preterm across Europe. The population-based sample was drawn from defined geographic areas rather than clinic-based populations; consequently, selection biases are unlikely to represent a major problem. It also used a validated generic measure of children’s HRQoL that facilitates comparisons with the HRQoL outcomes experienced by general childhood and clinical populations [48, 57]. Notably, a comprehensive analytical strategy was adopted to disentangle the direct and indirect effects of gestational age at birth on HRQoL outcomes.

Inevitably, there are limitations to this study. Firstly, the methods for collecting HRQoL data were heterogeneous across countries. For example, Denmark and Germany provided substantially lower numbers of observations due to the different schooling systems in those countries as registration for schooling starts later. To overcome this, a sensitivity analysis excluding these two countries was performed, the results of which showed a similar pattern. Secondly, the study is based on the untestable assumption of no omitted variables for a causal mediation analysis [58]. In order to minimise this potential bias, we adjusted for a broad range of covariates that can act as potential confounders in the multilevel OLS regression. Thirdly, this study was performed using parental reports of children’s HRQoL and there may be biases associated with parental reporting. There are a number of studies with respect to cross reporting of children’s HRQoL from both parents and children [59, 60], but they are mostly concentrated in children who can accurately express their thoughts and feelings (e.g. 8–16 years old) as pre-school aged children’s communicative expressions are generally constrained. In addition, a recent study revealed that five-year-old children could not independently provide PedsQL GCS data of sufficient psychometric quality [61]. As such, this is not a problem unique to this study.

Our findings offer insights into factors that affect the HRQoL of children born very or extremely preterm. We anticipate that these insights will be useful input for policy making. By understanding which physical or mental ailments influence the HRQoL that VPT born children experience, researchers, clinicians and decision-makers should be able to concentrate their efforts on proactively targeting children with those particular morbidities. In terms of the health research agenda, the data presented in this study can be used in applied work. For example, our findings should be of help to researchers aiming to reflect the differences in HRQoL associated with different characteristics of preterm born children in prognostic models. Our findings can further be used to target services towards higher risk groups with severe morbidities rather than all children born very preterm. The data reported in this study can also act as a significant new resource that can inform HRQoL estimation within the context of complications associated with preterm birth.

In conclusion, this study describes the magnitude and mechanisms of the HRQoL outcomes of children born very preterm based on a large cohort recruited from 11 European countries. Equally importantly, the study’s findings give rise to further questions that could trigger subsequent research on the topic. Based on our findings, further research is needed to further clarify the mechanisms through which different morbidities affect the HRQoL of children born very preterm.

## Supplementary Information

Below is the link to the electronic supplementary material.

Supplementary file1 (DOCX 23 kb)

## References

[1] D’Onofrio BM (2013). Preterm birth and mortality and morbidity: A population-based quasi-experimental study. JAMA Psychiatry.

[2] Arpino C (2010). Preterm birth and neurodevelopmental outcome: A review. Child’s nervous system.

[3] CDC. *Preterm birth.* 2021 [cited 21 Oct 2021]; Available from: https://www.cdc.gov/reproductivehealth/maternalinfanthealth/pretermbirth.htm.

[4] Moore, T., Hennessy, E. M., Myles, J., Johnson, S. J. Draper, E. S., Costeloe, K. L., Marlow, N. (2012) Neurological and developmental outcome in extremely preterm children born in England in 1995 and 2006: the EPICure studies. *BMJ**345*, e7961. 10.1136/bmj.e7961 PMC3514471 23212880

[5] Larroque B (2008). Neurodevelopmental disabilities and special care of 5-year-old children born before 33 weeks of gestation (the EPIPAGE study): A longitudinal cohort study. The Lancet.

[6] Serenius F (2013). Neurodevelopmental outcome in extremely preterm infants at 2.5 years after active perinatal care in Sweden. JAMA.

[7] Taylor HG (2011). Learning problems in kindergarten students with extremely preterm birth. Archives of pediatrics & adolescent medicine.

[8] Khan, K. A., Petrou, S., Dritsaki, M., Johnson, S. J., Manktelow, B., Draper, E. S., Smith, L. K., Seaton, S. E., Marlow, N., Dorling, J. (2015). Economic costs associated with moderate and late preterm birth: a prospective population‐based study. *BJOG: An International Journal of Obstetrics and Gynaecology*, 122(11):1495-1505 10.1111/1471-0528.13515 26219352

[9] Johnson S, Wolke D (2013). Behavioural outcomes and psychopathology during adolescence. Early Human Development.

[10] Saigal S, Doyle LW (2008). An overview of mortality and sequelae of preterm birth from infancy to adulthood. The Lancet.

[11] Petrou S, Krabuanrat N, Khan K (2020). Preference-based health-related quality of life outcomes associated with preterm birth: A systematic review and meta-analysis. PharmacoEconomics.

[12] Zwicker JG, Harris SR (2008). Quality of life of formerly preterm and very low birth weight infants from preschool age to adulthood: A systematic review. Pediatrics.

[13] van der Pal S (2020). Quality of life of adults born very preterm or very low birth weight: A systematic review. Acta Paediatrica.

[14] Olsen JE (2020). Early general movements are associated with developmental outcomes at 4.5–5 years. Early Human Development.

[15] Dvir Y (2019). Psychiatric symptoms: Prevalence, co-occurrence and functioning among extremely low gestational age newborns at age ten years. Journal of Developmental and Behavioral Pediatrics.

[16] Kelly MM (2014). Assessment of life after prematurity in 9-to 10-year-old children. MCN: The American Journal of Maternal/Child Nursing.

[17] Varni JW, Seid M, Kurtin PS (2001). PedsQL™4.0: reliability and validity of the pediatric quality of life inventory™version 4.0 generic core scales in healthy and patient populations. Medical Care.

[18] Thébaud B (2019). Bronchopulmonary dysplasia. Nature Reviews Disease Primers.

[19] Gou X (2018). Association between bronchopulmonary dysplasia and cerebral palsy in children: A meta-analysis. British Medical Journal Open.

[20] Darlow BA (2005). Prenatal risk factors for severe retinopathy of prematurity among very preterm infants of the Australian and New Zealand Neonatal Network. Pediatrics.

[21] Rogers B (1994). Cystic periventricular leukomalacia and type of cerebral palsy in preterm infants. The Journal of pediatrics.

[22] Patole S (2007). Prevention and treatment of necrotising enterocolitis in preterm neonates. Early Human Development.

[23] Theunissen NCM (2001). Quality of life in preschool children born preterm. Developmental Medicine and Child Neurology.

[24] Zeitlin J (2020). Cohort profile: Effective Perinatal Intensive Care in Europe (EPICE) very preterm birth cohort. International Journal of Epidemiology.

[25] PedsQL.org. *PedsQL™*. 2021 [cited 15 Aug 2021]; Available from: http://pedsql.org/index.html.

[26] Khan KA (2015). Economic costs associated with moderate and late preterm birth: a prospective population-based study. BJOG: An International Journal of Obstetrics and Gynaecology.

[27] Stylianou-Riga P (2018). Maternal socioeconomic factors and the risk of premature birth and low birth weight in Cyprus: A case–control study. Reproductive Health.

[28] Kwinta P, Pietrzyk JJ (2010). Preterm birth and respiratory disease in later life. Expert Review of Respiratory Medicine.

[29] Kim, S.W., Andronis L., Seppänen, A-V., Aubert, A. M., Zeitlin, J., Barros, H., Draper, E. S., Petrou, S., on behalf of the SHIPS Research Group. (2021). Economic costs at age five associated with very preterm birth: Multinational European cohort study. *Pediatric Research**122*(11), 1495–1505. 10.1038/s41390-021-01769-z PMC9556316 34773085

[30] Zeitlin J (2013). Preterm birth time trends in Europe: a study of 19 countries. BJOG: An International Journal of Obstetrics & Gynaecology.

[31] Zeitlin J (2017). Variation in term birthweight across European countries affects the prevalence of small for gestational age among very preterm infants. Acta Paediatrica.

[32] O'Reilly M, Sozo F, Harding R (2013). Impact of preterm birth and bronchopulmonary dysplasia on the developing lung: Long-term consequences for respiratory health. Clinical and Experimental Pharmacology and Physiology.

[33] MacKinnon DP, Fairchild AJ, Fritz MS (2007). Mediation analysis. J Annual Review of Psychology.

[34] Baron RM, Kenny DA (1986). The moderator–mediator variable distinction in social psychological research: Conceptual, strategic, and statistical considerations. J Journal of personality social psychology.

[35] VanderWeele T (2015). Explanation in causal inference: methods for mediation and interaction.

[36] StataCorp. Stata Structural Equation Modeling Reference Manual release 17. 2021 [cited 15 Aug 2021]; Available from: https://www.stata.com/manuals/sem.pdf.

[37] Rucker DD (2011). Mediation analysis in social psychology: Current practices and new recommendations. Social and Personality Psychology Compass.

[38] Lockwood, C.M., & MacKinnon, D.P. (1998). Bootstrapping the standard error of the mediated effect. In: *Proceedings of the 23rd annual meeting of SAS Users Group International*. Citeseer.

[39] Sriram, S., Schreiber, M. D., Msall, M. E., Kuban, K. C. K., Joseph, R. M., O’ Shea, T. M., Allred, E. N., Leviton, A., for the ELGAN Study Investigators. (2018). Cognitive development and quality of life associated with BPD in 10-year-olds born preterm. *Pediatrics**141*(6), e20172719. 10.1542/peds.2017-2719 PMC6317639 29773664

[40] Vederhus BJ (2010). Health related quality of life after extremely preterm birth: A matched controlled cohort study. Health and Quality of Life Outcomes.

[41] Brady JM (2019). Living with severe bronchopulmonary dysplasia—Parental views of their child’s quality of life. The Journal of Pediatrics.

[42] Joffe AR (2020). Kindergarten-age neurocognitive, functional, and quality-of-life outcomes after liver transplantation at under 6 years of age. Pediatric Transplantation.

[43] Varni JW (2003). The PedsQL™* 4.0 as a pediatric population health measure: feasibility, reliability, and validity. Ambulatory Pediatrics.

[44] Hilliard ME (2013). Identification of minimal clinically important difference scores of the PedsQL in children, adolescents, and young adults with type 1 and type 2 diabetes. Diabetes Care.

[45] Gire C (2019). Quality of life of extremely preterm school-age children without major handicap: A cross-sectional observational study. Archives of Disease in Childhood.

[46] Ni, Y., Johnson, S., Marlow, N., Wolke, D. (2021). Reduced health-related quality of life in children born extremely preterm in 2006 compared with 1995: the EPICure Studies. Archives of Disease in Childhood-Fetal and Neonatal Edition 10.1136/archdischild-2021-322888 PMC9209681 34697040

[47] Rautava L (2009). Health-related quality of life in 5-year-old very low birth weight infants. The Journal of Pediatrics.

[48] Berbis J (2012). Quality of life of early school-age French children born preterm: A cohort study. European Journal of Obstetrics & Gynecology and Reproductive Biology.

[49] Chien LY (2006). Health-related quality of life among 3–4-year-old children born with very low birthweight. Journal of advanced Nursing.

[50] Krieger N (2018). Severe sociopolitical stressors and preterm births in New York City: 1 September 2015 to 31 August 2017. Journal of Epidemiology and Community Health.

[51] Khanolkar AR (2015). Preterm and postterm birth in immigrant-and Swedish-born parents: A population register-based study. European Journal of Epidemiology.

[52] Vieira MEB, Linhares MBM (2016). Quality of life of individuals born preterm: A systematic review of assessment approaches. Quality of Life Research.

[53] Beaudoin S (2013). Healthcare utilization and health-related quality of life of adult survivors of preterm birth complicated by bronchopulmonary dysplasia. J Acta Paediatrica.

[54] Linsell, L., Malouf, R., Morris, J., Kurinczuk, J. J., Marlow, N. (2016). Prognostic factors for cerebral palsy and motor impairment in children born very preterm or very low birthweight: A systematic review. *Developmental Medicine & Child Neurology*, *58*(6):554-569 10.1111/dmcn.12972 PMC5321605 26862030

[55] Kenny D (2008). Reflections on mediation. J Organizational Research Methods.

[56] Kenny, D. *Mediation*. 2021 [cited 5 Nov 2021]; Available from: http://davidakenny.net/cm/mediate.htm.

[57] Palta M, Sadek-Badawi M (2008). PedsQL relates to function and behavior in very low and normal birth weight 2-and 3-year-olds from a regional cohort. Quality of Life Research.

[58] Yu Y (2019). Mediating roles of preterm birth and restricted fetal growth in the relationship between maternal education and infant mortality: A Danish population-based cohort study. PLoS medicine.

[59] Jozefiak T (2008). Quality of Life as reported by school children and their parents: A cross-sectional survey. Health Quality of Life Outcomes.

[60] Theunissen, N. C., Vogels, T. G., Koopman, H. M., Verrips, G. H., Zwinderman, K. A., Verloove-Vanhorick, S. P., Wit, J. M. (1998). The proxy problem: Child report versus parent report in health-related quality of life research. *Quality of Life Research*, *7*(5), 387–397. 10.1023/a:1008801802877 9691719

[61] Conijn JM, Smits N, Hartman EE (2020). Determining at what age children provide sound self-reports: An illustration of the validity-index approach. Assessment.

