# Supplementary material for: Health-related quality of life of children born very preterm: a multinational European cohort study
Source: Qual Life Res. 2022 Aug 17;32(1):47–58. doi: 10.1007/s11136-022-03217-9 (PMC9829588; doi:10.1007/s11136-022-03217-9)
Supplement: Supplementary file 1 — Supplementary file1 (DOCX 23 kb) [file 11136_2022_3217_MOESM1_ESM.docx]

# **Appendices**

**Appendix 1: Differences in PedsQL GCS scores by preterm status (four gestational categories)**

| **PedsQL GCS Score** |  | **<26 weeks** |  | **26-27 weeks** |  | **28-29 weeks** |  | **30-31 weeks** | **P-Value*** |
| --- | --- | --- | --- | --- | --- | --- | --- | --- | --- |
|  |  | **Mean (SD)** |  | **Mean (SD)** |  | **Mean (SD)** |  | **Mean (SD)** |  |
| Physical functioning |  | 75.33 (22.75) |  | 78.77 (22.48) |  | 79.97 (21.34) |  | 82.04 (19.92) | <0.001 |
| Emotional functioning |  | 73.10 (18.14) |  | 75.63 (17.54) |  | 76.50 (16.68) |  | 75.98 (16.74) | 0.0269 |
| Social functioning |  | 76.06 (21.14) |  | 80.36 (19.62) |  | 82.05 (19.46) |  | 83.12 (18.31) | <0.001 |
| School functioning |  | 69.41 (19.76) |  | 74.44 (19.44) |  | 75.00 (20.52) |  | 77.36 (18.51) | <0.001 |
| Psychosocial functioning |  | 73.11 (16.58) |  | 76.89 (15.66) |  | 78.03 (15.43) |  | 78.9 (14.69) | <0.001 |
| Total score |  | 73.35 (17.44) |  | 77.44 (15.94) |  | 78.53 (15.45) |  | 79.74 (14.67) | <0.001 |

* One way analysis of variance (ANOVA) test was used.

**Appendix 2: Multi-level regression analysis of the association between the total PedsQL GCS score and clinical and sociodemographic factors, excluding Denmark and Germany^1^**

|  |  |  | **Partially adjusted^+^** | **Fully adjusted** |
| --- | --- | --- | --- | --- |
| **Gestational age (weeks) (ref: 30-31)** | **<26** |  | -4.74 (2.47) | -0.40 (1.84) |
|  | **26-27** |  | -1.25 (0.92) | 0.57 (0.80) |
|  | **28-29** |  | -0.65 (0.97) | -0.24 (0.91) |
| **SGA (ref: ≥10th centile)** | **<10th centile** | | -0.87 (0.70) | -0.57 (0.66) |
| **Congenital anomalies (ref: no)** | **Yes** |  | -4.19 (0.89) *** | -3.43 (1.00) *** |
| **Sex (ref: female)** | **Male** |  | -3.07 (0.79) *** | -2.89 (0.77) *** |
| **Multiplicity (ref: singleton)** | **Twins and higher** |  | 1.98 (0.82) * | 1.84 (0.88) * |
| **BPD (ref: no)** | **Yes** |  |  | -3.68 (0.85) *** |
| **Severe non-respiratory neonatal morbidity (ref: no)** | **Yes** |  |  | -5.93 (1.21) *** |
| **Mother’s education (ref: higher education)** | **High school or less** | | -1.99 (0.62) ** | -1.78 (0.70) * |
| **Country of birth for mothers (ref: native)** | **Non-native, European born** | | -0.88 (1.57) | 0.12 (1.60) |
|  | **Non-native, non-European born** | | -5.92 (0.93) *** | -6.63 (0.95) *** |
| **Mother’s age at childbirth (years) (ref: 25-34)** | **<25** |  | -0.84 (1.04) | -0.59 (1.01) |
|  | **>34** |  | 1.59 (1.10) | 1.62 (1.15) |
| **Parity (ref: multiparous)**  **Primiparous** | |  | -0.20 (0.71) | -0.20 (0.61) |

1: Multi-level regression analysis was performed. The following levels of the multi-level analysis were specified: 1) individual child, 2) parent, and 3) country.

^+^: The partially adjusted model excludes BPD and severe non-respiratory morbidity.

SGA: small for gestational age

BPD: bronchopulmonary dysplasia

Severe non-respiratory: Intraventricular haemorrhage grades III-IV (IVH), periventricular leukomalacia (PVL), retinopathy of prematurity stages III-V (ROP) or necrotising enterocolitis needing surgery (NEC)

| *p<0.05 | **p<0.01 | ***p<0.001 |
| --- | --- | --- |

**Appendix 3: Multi-level regression analysis of the association between the total PedsQL GCS score and clinical and sociodemographic factors (gestational age as a continuous variable)**

|  |  |  | **Fully adjusted**  **Coef (SE)** |
| --- | --- | --- | --- |
| **Gestational age (weeks)** |  |  | -0.004 (0.16) |
| **SGA (ref: ≥10th centile)** | **<10th centile** | | -0.44 (0.60) |
| **Congenital anomalies (ref: no)** | **Yes** |  | -4.16 (1.01) *** |
| **Sex (ref: female)** | **Male** |  | -2.34 (0.55) |
| **Multiplicity (ref: singleton)** | **Twins and higher** | | 2.14 (1.48) |
| **BPD (ref: no)** | **Yes** |  | -3.64 (0.89) *** |
| **Severe non-respiratory morbidity (ref: no)** | **Yes** |  | -6.19 (0.97) *** |
| **Mother’s education (ref: higher education)** | **High school or less** | | -1.59 (0.58) |
| **Country of birth for mothers (ref: native)** | **Non-native, European born** | | -1.8 (1.36) |
|  | **Non-native, non-European born** | | -5.73 (0.91) *** |
| **Mother’s age at childbirth (years) (ref: 25-34)** | **<25** |  | 0.32 (0.95) |
|  | **>34** |  | 0.23 (0.63) |
| **Parity (ref: multiparous)** | **Primiparous** | | 0.75 (0.59) |

SGA: small for gestational age

BPD: bronchopulmonary dysplasia

Severe non-respiratory: Intraventricular haemorrhage grades III-IV (IVH), periventricular leukomalacia (PVL), retinopathy of prematurity stages III-V (ROP) or necrotising enterocolitis needing surgery (NEC)

| *p<0.05 | **p<0.01 | ***p<0.001 |
| --- | --- | --- |

**Appendix 4: Generalised Structural Equation Modelling (GSEM) of the effects of preterm status on the total PedsQL GCS score mediated through BPD and severe non-respiratory morbidity**

|  |  | **Coef (SE)** |
| --- | --- | --- |
| **Gestational age (weeks) (ref: 30-31)** | **<26** | -1.03 (1.19) |
|  | **26-27** | 0.06 (0.80) |
|  | **28-29** | -0.44 (0.67) |
| **SGA (ref: ≥10th centile)** | **<10th centile** | -0.42 (0.60) |
| **Congenital anomalies (ref: no)** | **Yes** | -4.11 (1.02) *** |
| **Sex (ref: female)** | **Male** | -2.34 (0.55) *** |
| **Multiplicity (ref: singleton)** | **Twins and higher** | 1.84 (0.60) ** |
| **BPD (ref: no)** | **Yes** | -3.57 (0.90) *** |
| **Severe non-respiratory morbidity (ref: no)** | **Yes** | -6.02 (0.98) *** |
| **Mother’s education (ref: higher education)** | **High school or less** | -1.57 (0.58) ** |
| **Country of birth for mothers (ref: native)** | **Non-native, European born** | -1.67 (1.36) |
|  | **Non-native, non-European born** | -5.70 (0.92) *** |
| **Mother’s age at childbirth (years) (ref: 25-34)** | **<25** | 0.27 (0.95) |
|  | **>34** | 0.18 (0.63) |
| **Parity (ref: multiparous)** | **Primiparous** | 0.82 (0.59) |

SGA: small for gestational age

BPD: bronchopulmonary dysplasia

| *p<0.05 | **p<0.01 | ***p<0.001 |
| --- | --- | --- |

**Appendix 5:** **Sensitivity analysis that excludes Denmark and Germany from the mediation analysis** **on the total PedsQL GCS score (four categories of gestational age: <26 weeks, 26-27 weeks; 28-29 weeks; 30-31 weeks (referent))^1^**

|  |  | **Coef (SE)** | **Bootstrap 95% confidence interval** |
| --- | --- | --- | --- |
| **<26** | BPD: indirect | -1.84 (0.49) | (-2.81, -0.88) |
|  | Direct² | -0.70 (1.35) | (-3.34, 1.94) |
|  | BPD: total | -2.54 (1.35) | (-5.19, 0.10) |
|  | Severe non-respiratory morbidity: indirect | -18.16 (3.68) | (-25.37, -10.96) |
|  | Direct² | -0.70 (1.35) | (-3.34, 1.94) |
|  | Severe non-respiratory morbidity: total | -18.86 (3.58) | (-25.88, -11.84) |
| **26-27** | BPD: indirect | -1.12 (0.3) | (-1.7, -0.53) |
|  | Direct² | 0.43 (0.83) | (-1.19, 2.05) |
|  | BPD: total | -0.68 (0.83) | (-2.32, 0.95) |
|  | Severe non-respiratory morbidity: indirect | -11.33 (2.32) | (-15.88, -6.78) |
|  | Direct² | 0.43 (0.83) | (-1.19, 2.05) |
|  | Severe non-respiratory morbidity: total | -10.90 (2.34) | (-15.49, -6.31) |
| **28-29** | BPD: indirect | -0.38 (0.11) | (-0.59, -0.16) |
|  | Direct² | -0.36 (0.71) | (-1.74, 1.03) |
|  | BPD: total | -0.73 (0.72) | (-2.14, 0.67) |
|  | Severe non-respiratory morbidity: indirect | -4.87 (1.23) | (-7.27, -2.46) |
|  | Direct² | -0.36 (0.71) | (-1.74, 1.03) |
|  | Severe non-respiratory morbidity: total | -5.22 (1.43) | (-8.02, -2.43) |

^1^: This was performed based on 1000 bootstrap simulations.

²: This represents the direct effect of gestational age (GA) on the PedsQL GCS score
